# Bilateral optic perineuritis: a rare manifestation of giant cell arteritis - case report and literature review

**DOI:** 10.3389/fopht.2025.1598302

**Published:** 2025-07-04

**Authors:** Jnadi Madkhali, Ahmed B. Aba Alkhail, Mohammed A. Aldriweesh, Yaser Al Malik

**Affiliations:** ^1^ Department of Neurology, King Abdulaziz Medical City, Ministry of National Guard-Health Affairs, Riyadh, Saudi Arabia; ^2^ King Abdullah International Medical Research Center, Riyadh, Saudi Arabia; ^3^ College of Medicine, King Saud bin Abdulaziz University for Health Sciences, Riyadh, Saudi Arabia

**Keywords:** optic perineuritis, vision loss, giant cell arteritis, brain imaging, case report

## Abstract

**Background:**

Giant cell arteritis (GCA) is a granulomatous vasculitis in older individuals that primarily affects medium-to-large vessels. Owing to the involvement of the ophthalmic arteries, GCA can lead to severe ischemic complications, including vision loss. Optic perineuritis (OPN) is characterized by inflammation around the optic nerve sheath and is a rare manifestation of GCA with diagnostic and therapeutic challenges.

**Case presentation:**

This case study reports a 75-year-old female presenting with subacute constitutional symptoms of fever and poor appetite with bilateral eye pain and visual disturbance. The patient had elevated inflammatory markers, specifically an erythrocyte sedimentation rate of 120 mm/h, with imaging findings consistent with bilateral OPN and temporal artery biopsy-proven GCA. Treatment with high-dose dexamethasone, followed by oral prednisolone and tocilizumab, led to symptomatic improvement in vision stabilization.

**Conclusion:**

This case highlights the importance of recognizing OPN as a possible manifestation of GCA. Although cases of OPN are mostly idiopathic, it can rarely represent the first manifestation of GCA. Therefore, prompt diagnosis through brain imaging and temporal artery biopsy is essential, and aggressive treatment with steroids is crucial for managing GCA-associated OPN to prevent irreversible vision loss.

## 1 Introduction

Giant cell arteritis (GCA) is the predominant primary systemic vasculitis in adults, mostly affecting individuals aged 50 and older (1). In a notable proportion (25–50% of cases), GCA induces ocular manifestations through inflammatory constriction of the ophthalmic and short posterior ciliary arteries that supply blood to the optic nerve and nerve head (2). The most common cause is arteritic anterior ischemic optic neuropathy, which primarily arises from optic disc ischemia, followed by arteritic posterior ischemic optic neuropathy caused by retrobulbar injury (3, 4). Less frequent etiologies include central retinal and cilioretinal artery occlusion. However, visual impairments attributable to optic perineuritis (OPN) are exceptionally rare, with only a limited number of cases documented in the literature. The significance of this case lies in its presentation of isolated bilateral OPN in a 75-year-old woman with GCA and no disc edema, a manifestation seldom reported and often challenging to diagnose. This case contributes to the growing recognition of atypical ocular complications in GCA, underscoring the need for increased clinical awareness and early detection.

## 2 Case description

A 75-year-old female with a history of pulmonary hypertension, dyslipidemia, bronchiectasis, and grade II diastolic heart failure presented to the emergency department. She had a 3-week history of bilateral eye pain followed by blurred vision, a documented fever at home of 38 degrees, new onset persistent mild-to-moderate holocephalic headache, and poor appetite. The patient initially visited multiple hospitals and was diagnosed with glaucoma and sinusitis. The patient underwent minimally invasive laser surgery and was discharged on oral augmentin without any reportable improvement.

The patient did not have any other neurological symptoms, such as recurrent headache attacks, double vision, weight loss, painful eye movements, limb numbness or weakness, joint pain, or jaw claudication. Upon arrival at the emergency department, the patient had a low-grade fever of 38.2 degrees with normal blood pressure and heart rate. Her initial neurological examination was nonfocal, with no meningeal signs or encephalitis. Ophthalmological examination revealed bilateral early cataracts and a small optic disc on both sides with a cup-to-disc ratio of 0.3, normal color vision, and no evidence of a relative afferent pupillary defect. Her visual acuity was 20/60 on both sides, with no evidence of disc edema. Her macula was healthy, and the intraocular pressure was low; therefore, the anti-glaucoma drops were discontinued. Systemic examination was unremarkable, and the temporal artery was palpable on both sides, with no tenderness.

Initial laboratory tests showed leukocytosis of 13,000 cells, a hemoglobin level of 11 gram/dL, an erythrocyte sedimentation rate (ESR) of 120 mm/h, and a C-reactive protein (CRP) level of 141 mg/L (normal lab value <3). Serum electrolyte, liver, and renal function test results were normal. Non-contrast brain computed tomography (CT) did not reveal acute or chronic central nervous system insults or space-occupying lesions. Meningitis was suspected, considering the history of headache with fever, and a lumbar puncture was performed, which was unremarkable with normal cerebrospinal fluid protein, glucose, cell count, culture, and viral multiplex. The patient was started on empirical Piperacillin/Tazobactam (Tazocin^®^) and admitted for further investigation as a case of fever of unknown origin.

Her systemic workup was negative for brucellosis, tuberculosis, cytomegalovirus, Epstein–Barr virus, hepatitis sexology, human immunodeficiency virus, and malaria. No organisms were identified in blood, urine, or respiratory cultures. Moreover, vasculitis workup, including anti-nuclear antibodies, anti-double-stranded antibodies, Anti-Sjögren’s-syndrome-related antigen A (Ro) and Anti-Sjögren’s-syndrome-related antigen B (La), antineutrophilic cytoplasmic antibodies, complement levels, cyclic citrullinated peptide antibodies, and rheumatoid factor levels, were within normal ranges. Serum folate, vitamin B12, thyroid-stimulating hormone, and parathyroid hormone levels were normal. Chest and abdominal CT revealed no suspicious infectious or inflammatory processes.

**Figure 1:**
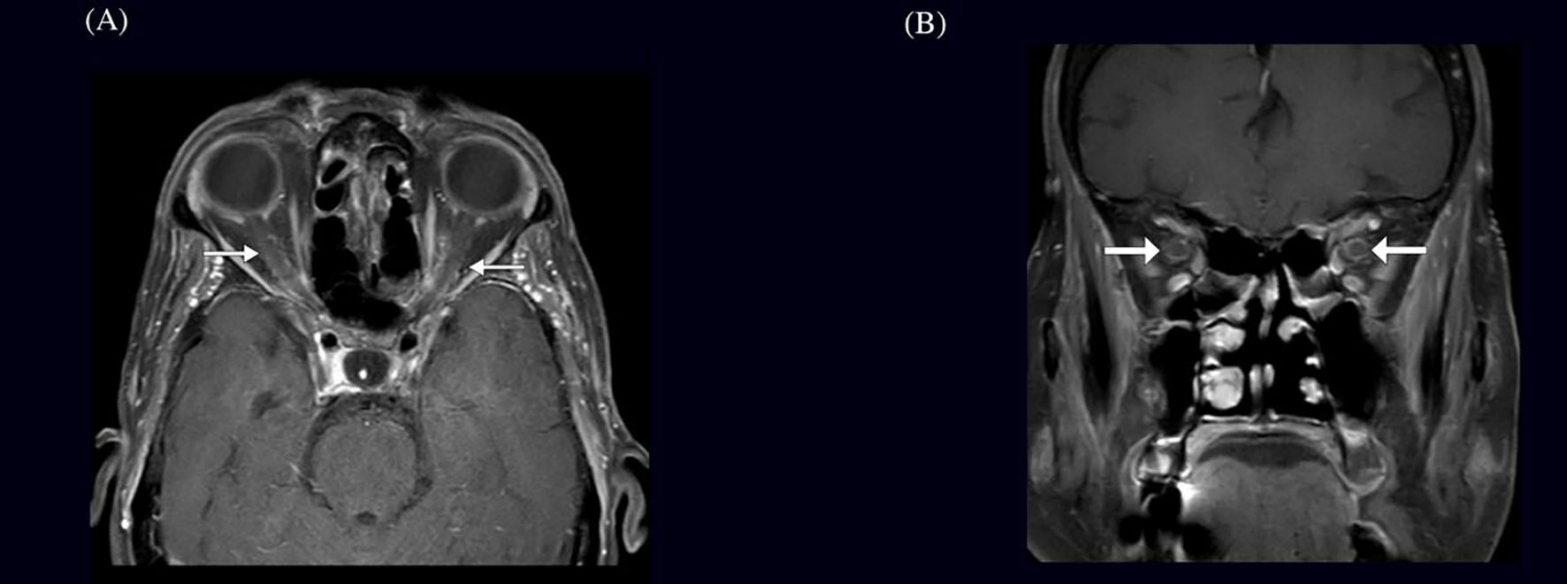
MRI findings in a patient with bilateral optic nerve perineuritis **(A)** Axial and **(B)** Coronal brain MRI showing post-contrast T1-weighted image with fat suppression demonstrating bilateral optic nerve sheath enhancement, characteristic of perineuritis.

**Figure 2:**
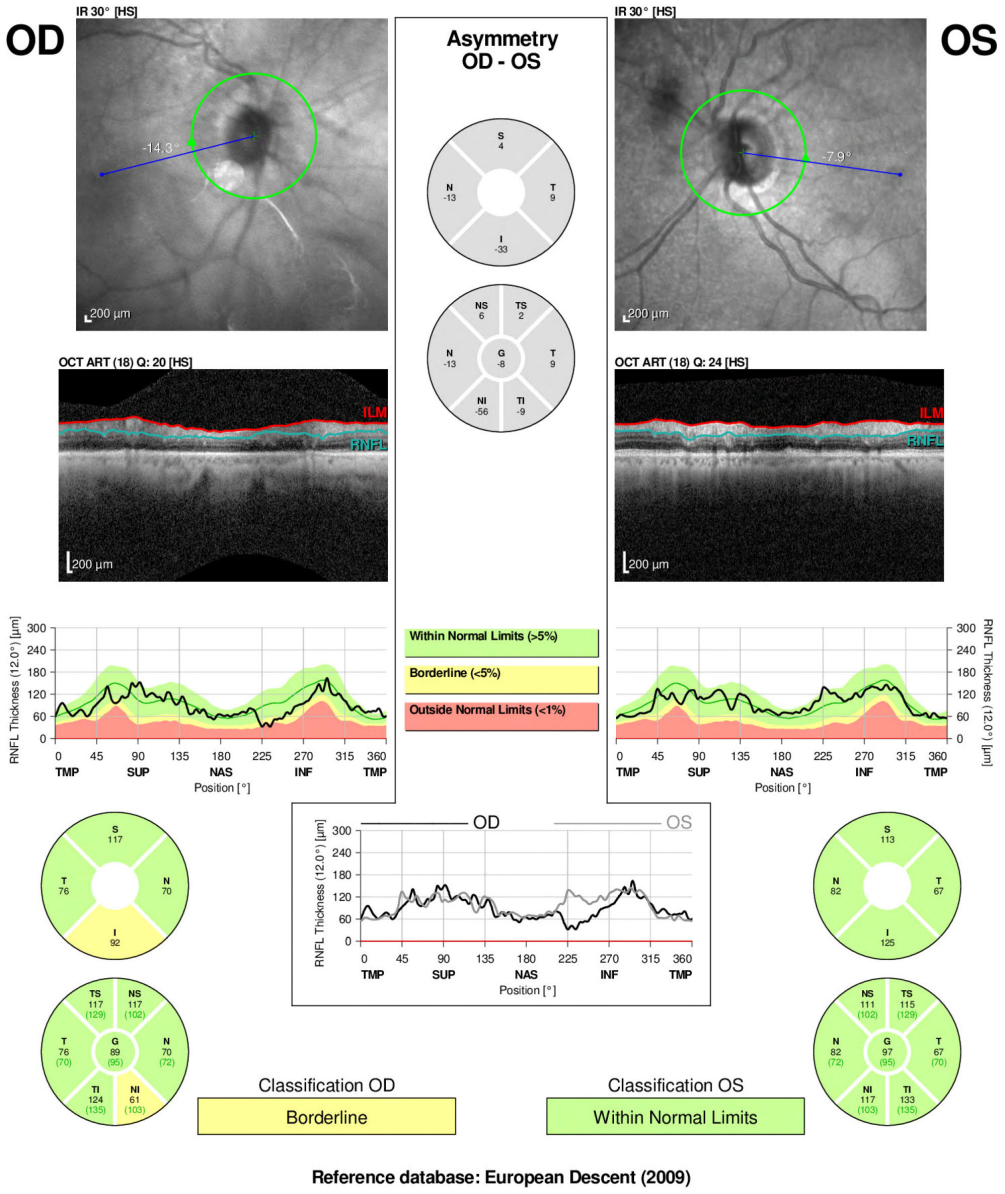
Optical coherence tomography (OCT) and retinal nerve fiber layer (RNFL) analysis of both eyes in a patient with optic perineuritis done 3 weeks after start on symptoms. Infrared imaging and cross-sectional OCT scans demonstrate optic disc appearance and RNFL thickness. The right eye (OD) shows borderline RNFL thinning, particularly in the nasal and inferior quadrants, whereas the left eye (OS) remains within normal limits.

**Figure 3:**
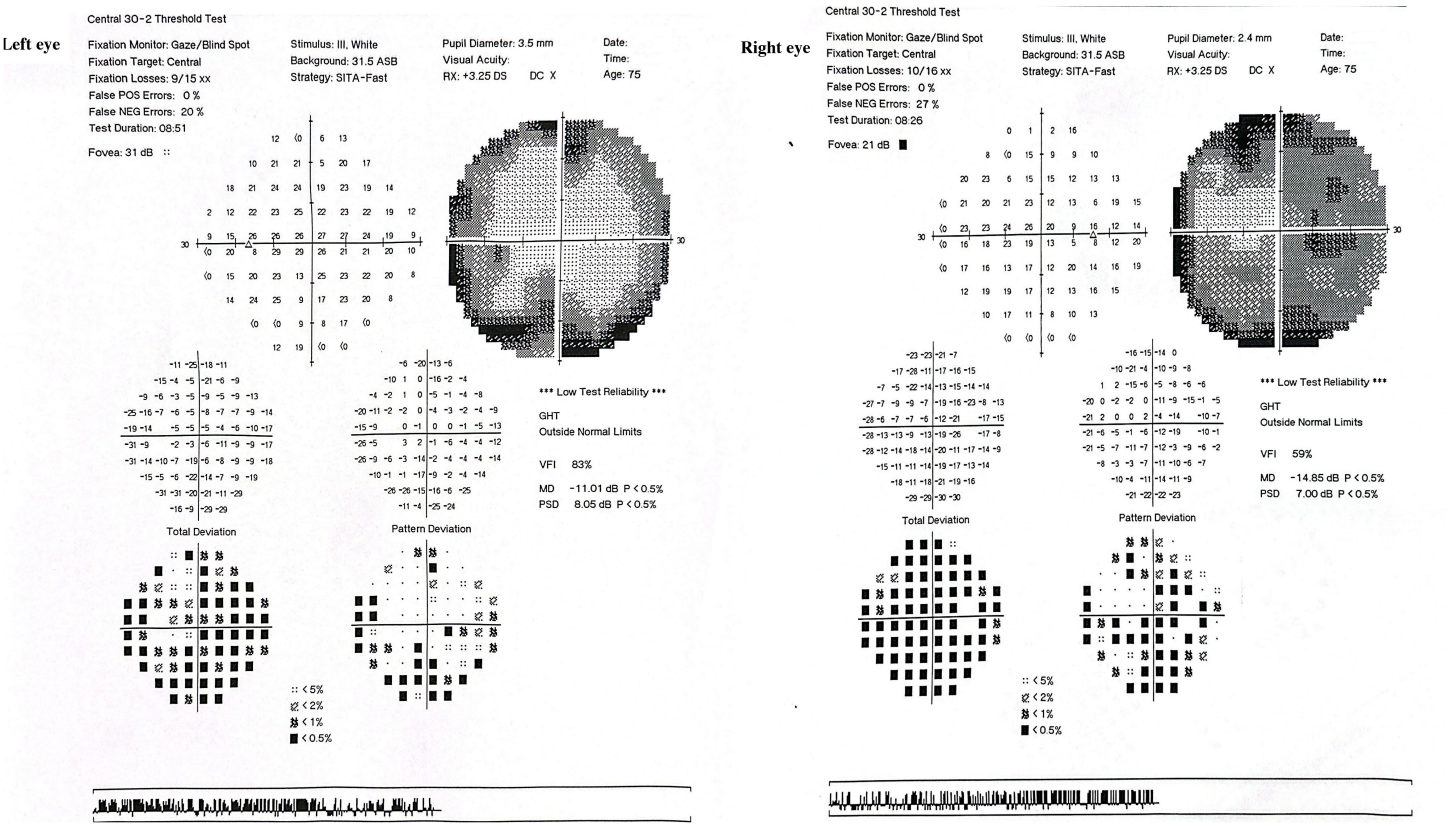
Humphrey Visual Field (HVF) 30–2 threshold tests of both eyes The right eye (OD) shows a mean deviation (MD) of −14.85 dB and visual field index (VFI) of 59%, with dense superior and peripheral field loss, particularly nasally, consistent with optic nerve dysfunction. The left eye (OS) demonstrates a mean deviation of −11.01 dB and VFI of 83%, with similar, though less severe, nasal and arcuate defects.

Brain and contrast-orbital magnetic resonance imaging (MRI) showed bilateral optic nerve sheath enhancements sparing the optic nerve extending to the chiasm and pituitary stalk without intracranial, meningeal, or parenchymal abnormalities (**Figure 1**). Findings of ophthalmic imaging studies are shown in **Figures 2**, **3**. Serum aquaporin 4 and myelin oligodendroglial cell antibodies for neuromyelitis optica spectrum disorder, and myelin oligodendrocyte glycoprotein

were negative; therefore, GCA was suspected, and a right temporal artery biopsy was performed.

**Figure 4:**
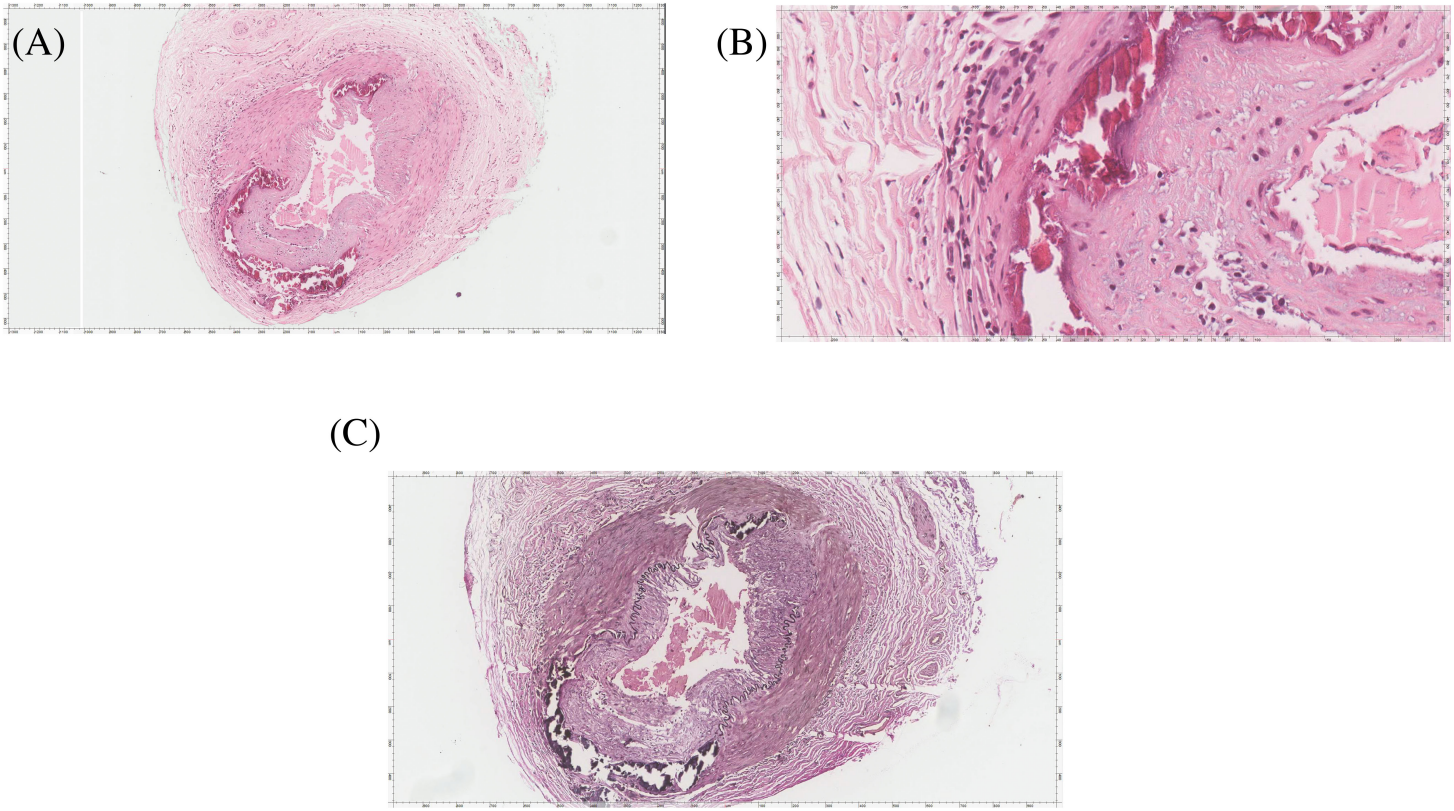
The temporal artery biopsy. **(A)** HE stained slide shows a muscular artery with lumen narrowing, intimal thickening, disruption of internal elastica, lymphoplasma histiocytic aggregates, and dystrophic calcification. **(B)** High magnification image highlighting the lymphoplasma histiocytic aggregates and dystrophic calcification. The disruption of internal elastica is best seen with the Elastic Verhoeff Van Gieson Stain in **(C)**.

The patient was started on empirical intravenous dexamethasone 60 mg three times on the same day as the procedure, and the biopsy results were positive for temporal artery luminal narrowing with intimal thickening, internal elastic layer disruption, lymphoblastic histiocytic aggregates, and dystrophic calcification, consistent with GCA (**Figure 4**). Two days after steroid treatment initiation, the patient reported marked improvement in headache and eye pain.

The patient was discharged on oral prednisolone 60 mg/day, with complete symptom resolution. Ten days after discharge, the patient was evaluated in a rheumatology clinic where weekly tocilizumab injections were started, and her prednisolone dose was tapered gradually with a plan to stay at 20 mg once daily. Two months later, the patient visited the follow-up clinic and was completely asymptomatic with a stable visual acuity of 20/40 in both eyes.

## 3 Discussion

Giant cell arteritis (GCA) is a systemic vasculitis of large and medium-sized vessels primarily affecting individuals over the age of 50. Its classic ophthalmologic manifestation, arteritic anterior ischemic optic neuropathy (AAION), is typically associated with disc edema due to ischemia of the optic nerve head. However, optic perineuritis (OPN) an inflammatory process confined to the optic nerve sheath is a rarely reported ocular manifestation of GCA, and even more uncommon when it presents bilaterally without disc edema.

Our patient represents a unique presentation of biopsy-proven GCA, manifesting as bilateral optic perineuritis without optic disc swelling or relative afferent pupillary defect. Magnetic resonance imaging confirmed optic nerve sheath enhancement sparing the optic nerve proper, consistent with OPN. This imaging profile, coupled with systemic inflammatory markers and constitutional symptoms, raised suspicion for GCA, which was subsequently confirmed histopathologically.

This case aligns with a small but growing body of literature documenting GCA-associated OPN (**Table 1**). Emami et al. described a 60-year-old African male with biopsy-proven GCA and bilateral optic nerve sheath enhancement. Although that patient demonstrated mild optic disc swelling and cotton wool spots, his visual acuity was preserved and systemic inflammatory markers were markedly elevated features shared with our case (5). Liu et al. also described a patient with GCA who exhibited bilateral optic nerve sheath enhancement on MRI without evidence of disc edema, underscoring the potential for GCA to present with isolated perineural inflammation (6)

**Table 1:** Comparison of current case with previous studies.

Feature	Current case	Emami et al., 2021 (5)	Albarrak et al., 2018 (7)	Liu et al., 2013 (6)
Age/sex	75/Female	60/Male	80/Male	83/Female, 68/Female
Initial symptoms	Bilateral eye pain, blurred vision, fever, headache, poor appetite	Left-sided headache, blurred vision, fatigue, weight loss, jaw claudication	Bilateral painless visual loss, headache	Headache, blurred vision, visual loss
Medical history	Pulmonary hypertension, bronchiectasis, grade II diastolic heart failure	Unremarkable	Hypertension, Type 2 Diabetes Mellitus	Unremarkable
Initial misdiagnosis	Glaucoma, sinusitis	Sinusitis	None	Non-arteritic anterior ischemic optic neuropathy
ESR (mm/h)	120	128	40	36, 10
CRP (mg/L)	141	91	132	NA, 1.1
Visual acuity	20/60 (both eyes)	20/20 (both eyes)	No light perception (both eyes)	Hand motions (OD), 20/30 (OS);20/20 (OD),20/25 (OS)
Fundoscopy/ophthalmoscopy	Bilateral early cataract, small optic disc, normal color vision, no relative afferent pupillary defect, no papilledema	Right optic nerve head mildly elevated, cotton wool spots	Normal discs and retina	Normal fundus, then pale optic disc
MRI findings	Bilateral optic nerve sheath enhancements	Bilateral optic nerve sheath enhancement	Lesion in right optic nerve suggestive of acute infraction	Bilateral perineural optic nerve enhancement
Temporal artery biopsy	Positive	Positive	Positive	Positive
Initial treatment	Intravenous Dexamethasone followed by oral prednisolone tapering dose and tocilizumab initiated	Intravenous Prednisone, then oral Prednisone, methotrexate	Intravenous methylprednisolone, then Prednisolone	Intravenous Methylprednisolone, then Prednisone
Final diagnosis	Giant cell arteritis	Giant cell arteritis	Giant cell arteritis	Giant cell arteritis
Outcome	Symptomatic improvement with steroids	Stable visual acuity, systemic symptoms resolved	No improvement in vision, headache improved	No light perception in both eyes, vision stable after treatment

Gold and Galetta documented a patient with sequential optic perineuritis followed by GCA diagnosis, suggesting that OPN may precede or even signal the onset of systemic vasculitis (8). Similarly, Pappolla et al. presented a case where bilateral OPN was the initial manifestation of GCA, emphasizing that perineural inflammation can occur without classic features such as disc edema or visual field defects (9).

The mechanism underlying OPN in GCA remains uncertain. One theory posits inflammation of the perineural pial vessels or vasa nervorum, in contrast to AAION where posterior ciliary artery occlusion is central. MRI findings in OPN typically reveal sheath enhancement with preserved axonal structure, as seen in our patient, and may reflect an early or isolated inflammatory process prior to ischemic damage (10).

This case shows the critical need for a structured diagnostic approach in elderly patients presenting with optic perineuritis, particularly when disc edema is absent. The presence of bilateral optic nerve sheath enhancement on MRI, systemic inflammatory markers, and constitutional symptoms should prompt strong consideration of GCA even in the absence of visual acuity loss or classic cranial features. Differential diagnoses such as infectious optic neuritis, sarcoidosis, neuromyelitis optica, and orbital inflammation must be excluded through targeted serological and radiologic evaluations. Temporal artery biopsy remains the gold standard, although high-resolution imaging modalities like black-blood MRI and vascular ultrasound have proven valuable in detecting inflammation in less accessible arteries like the occipital branch (11, 12). Management should be prompt and aggressive: intravenous corticosteroids followed by oral tapering, supplemented by tocilizumab or other steroid-sparing agents when indicated (11). Early recognition and intervention are essential to prevent irreversible visual sequelae and systemic complications.

OPN is a rare and unusual neuro-ophthalmic presentation of GCA. A high degree of suspicion can lead to timely diagnosis, prompt treatment and prevention of vision loss, as OPN typically responds well to corticosteroid therapy, as evidenced by our patient’s rapid improvement in visual symptoms and headache following high-dose steroids and tocilizumab.

## 4 Patient perspective

When I developed this eye pain and blurred vision in one eye, the first thing I thought about was raised intraocular pressure (glaucoma). When I visited a private center, the doctor there started me on anti-glaucoma drops for suspected glaucoma. A few days later, the vision continued to worsen and involved the other eye, along with a new-onset headache. I visited another doctor who told me it might be sinusitis. I was given antihistamines and a nasal decongestant, but again, there was no improvement. Finally, after two weeks, I visited your hospital, and the workup revealed giant cell arteritis. I was started on steroid therapy. I was afraid of losing my vision permanently; however, I am very grateful and thankful for all of your efforts, and I am doing fine right now with very good visual acuity.

## Data Availability

The datasets presented in this article are not readily available because of ethical and privacy restrictions. Requests to access the datasets should be directed to the corresponding author/s.
